# Working Conditions Influencing Drivers’ Safety and Well-Being in the Transportation Industry: “On Board” Program

**DOI:** 10.3390/ijerph181910173

**Published:** 2021-09-28

**Authors:** Susan E. Peters, Harrison Grogan, Gesele M. Henderson, María Andrée López Gómez, Marta Martínez Maldonado, Iván Silva Sanhueza, Jack T. Dennerlein

**Affiliations:** 1 Harvard T.H. Chan School of Public Health, Boston, MA 02115, USA; groganhm@mail.com (H.G.); j.dennerlein@northeastern.edu (J.T.D.); 2 Dana Farber Cancer Institute, Boston, MA 02115, USA; 3 Bouvé College of Health Sciences, Northeastern University, Boston, MA 02115, USA; ghenderson1@une.edu; 4 College of Osteopathic Medicine, University of New England, Biddeford, ME 04005, USA; 5 Sociology Department, Memorial University of Newfoundland, St. John’s, NL A1B 1T5, Canada; malopezgomez@mun.ca; 6 Mutual de Seguridad CChC, Santiago 8320000, Chile; mamartinez@mutual.cl (M.M.M.); isilvas@uchile.cl (I.S.S.)

**Keywords:** healthy work design and well-being, organizational design, healthy leadership, occupational stress, fatigue, bus driver, health promotion, Total Worker Health, focus groups, scheduling

## Abstract

The conditions of work for professional drivers can contribute to adverse health and well-being outcomes. Fatigue can result from irregular shift scheduling, stress may arise due to the intense job demands, back pain may be due to prolonged sitting and exposure to vibration, and a poor diet can be attributed to limited time for breaks and rest. This study aimed to identify working conditions and health outcomes in a bussing company by conducting focus groups and key informant interviews to inform a Total Worker Health^®^ organizational intervention. Our thematic analysis identified three primary themes: lack of trust between drivers and supervisors, the scheduling of shifts and routes, and difficulty performing positive health behaviors. These findings demonstrate the value of using participatory methods with key stakeholders to determine the unique working conditions and pathways that may be most critical to impacting safety, health, and well-being in an organization.

## 1. Introduction

Professional drivers, including bus, taxi, and long-haul truck drivers, experience higher rates of health issues compared to other occupations [1]. They suffer from acute and chronic health conditions at higher rates than the general working population, including increased risk of cardiovascular disease [2,3,4], obesity [5,6], and musculoskeletal disorders [1,7,8,9,10], and are more stressed [11,12]. Compared to the general population, professional drivers smoke more, consume higher amounts of alcohol, exercise less and access healthcare less [13,14]. Driver fatigue—caused by long shifts and tight schedules—has been found to result in a higher likelihood of accidents and fatalities on the road [15]. Irregular working schedules of professional drivers often impact their mental health, resulting in depression or anxiety [11,12].

It has been well established that work is a major determinant of health and well-being [16]. The working conditions professional drivers are exposed to day-to-day can have detrimental impacts on their safety, health, and well-being; and are characterized by high physical, psychosocial and organizational hazards, such as high workloads, the need for high levels of mental alertness, erratic and long work schedules, time pressures, exposure to chemicals such as fumes, disrupted sleep patterns, social isolation and loneliness, low job control, customer confrontations, as well as the standard risks of being a driver, such as the sedentary nature of driving, and the exposure to vibration through the seat of the vehicle and steering wheel [11,17,18,19,20,21,22,23,24]. These risks have been linked with various health conditions and have long-term ramifications on the overall well-being of drivers. The pathways for worker injury and ill-health share common root causes through these conditions of work [25]. Interventions that focus on changing working conditions to improve drivers’ safety, health and well-being are largely understudied, yet an important area of inquiry considering the large safety and health impacts that their work can expose them to. There is a shift in both research and practice in support of Total Worker Health^®^ (TWH) interventions that integrate both health protection and health promotion to target the conditions of work through upstream organizational approaches—workplace policies, programs, and practices—to address health and well-being disparities [26]. The goal of TWH interventions are to create safer and healthier work and work environments and advance worker safet, health, and well-being.

Various professional bus driver occupations differ in how their working conditions manifest, based on how their work is structured and by the physical, cognitive, and psychosocial demands required of them. For example, long-haul bus drivers tend to experience longer work hours and monotonous driving conditions when compared with city bus drivers, while city bus drivers often handle a greater intensity of demands due to higher traffic density, more frequent stops, and stressful passenger interactions. Some types of drivers experience a combination of factors experienced by both long-haul and city bus drivers, such as those working as charter or interurban bus drivers. Interventions designed to improve the health of professional drivers must therefore consider the work organization and demands, and how these demands may differ depending on their job and organization.

While industry-specific guidance for professional drivers can elucidate potential risks to workers’ occupational safety and health for an organization, the unique root cause for the many and varied safety, health, and well-being issues that drivers face can be difficult to precisely identify [4]. The determination of the root causes of risks to drivers’ safety, health, and well-being can be facilitated by working with all stakeholders of the organization to gain a more complete understanding of the factors that might contribute to drivers’ safety, health, and well-being using participatory methods. While quantitative surveys alone can be used to determine what is a strength or a challenge for an organization, they sometimes fail to provide the nuances needed to explain how working conditions, through an organization’s unique pathways, influence drivers’ health and safety behaviors, and their safety, health, and well-being [27]. Because of this, qualitative methods have been recognized as an effective tool to determine the factors that working conditions and other contextual factors have on workers’ safety, health, and well-being [28]. These data can then be used for intervention development using participatory methods involving the organization. Previous research with construction workers [29], food service workers [28], and grocery store workers [30] has demonstrated the effectiveness of such an approach.

The significance of this study is twofold. First, the findings of this study will be used to inform and tailor the design of a *TWH* intervention; specifically, the identification of the content mechanisms or working conditions that will be the targets for the intervention, and how these are percieved to influence drivers’ safety, health, and well-being. While it is part of a larger study that will develop and evaluate a TWH intervention—“*On Board Program*”,this research is necessary as not all working conditions manifest in the same way in companies in the same sector due to their unique characteristics. This will become especially important in the future as workplaces continue to pivot and expand to meet new markets, technologies and demands, and accommodate challenges such as the COVID-19 pandemic, financial crises, or social or political unrest [31]. Using employer-centered participatory methods focusing on upstream integrated approaches has been found to be effective in improving workers’ safety, health, and well-being [32,33]. Tailoring an intervention to fit the company is an important step to ensure its success and sustainability. This paper’s significance is grounded in a description of how we used replicable qualitative methods to identify working conditions and develop a conceptual model to inform the intervention design of the *On Board Program*; both researchers and practitioners may find this useful when developing *TWH* interventions. Second, there is little research on how working conditions can influence the safety, health, and well-being of drivers using a framework that considers the psychosocial work environment (e.g., supervisor support, coworker support, customer interactions, and isolation while on the road) and the organization of work (e.g., routes and scheduling and how these factors are intertwined to create considerable safety and health issues).

Thus, the purpose of this study was to (a) identify how working conditions influenced bus drivers’ safety, health, and well-being using qualitative methods and (b) develop a unique organization-centered conceptual model of theorized pathways for this organization through which working conditions influence workers’ safety, health, and well-being behaviors, and identify outcomes to be used in a future *TWH* intervention.

## 2. Materials and Methods

This study was the first phase of the *On Board Program* to develop a Total Worker Health^®^ intervention for drivers’ safety, health, and well-being. This intervention will later be implemented and evaluated in the second phase of this Project. The first phase of the *On Board Program* used qualitative methods based on grounded theory principles [34,35], and content analysis [36] to create an exploratory model to theorize how the conditions of work and work organizational factors influence drivers’ safety, health, and well-being. Using these principles, one examines concepts and interrelationships between concepts to show how and why a phenomenon occurs—such as the relationship between working conditions and drivers’ safety, health, and well-being. Capturing variability and trying to understand why variability exists was also important, lending itself to the grounded theory method.

Data were derived from two main sources: interviews with key company personnel and focus groups with drivers. These activities were conducted in late 2018. A model was then developed, based on analysis of these qualitative data.

### 2.1. Study Context

This study was conducted in collaboration with one medium-sized (450 drivers) privately owned bus transportation organization in Chile. This transportation company was selected because it was a customer of our Chilean collaborators Mutual de Seguridad, CChC, was one of two companies that had participated in research with academic collaborators previously, and its leadership had indicated an interest in implementing a TWH intervention aiming to improve the safety, health, and well-being of their drivers. The bus drivers were all employed by a single transportation organization located in a regional urban center. The organization was contracted by another company (“host company”) to transport workers to a remote worksite. The contractual nature of the agreement between the two companies was renewed every 2–3 years for services to transport workers and sub-contractors of the host company to the remote worksite for multiple shifts daily. Thus, workers live in their own urban communities and are picked up before and dropped off after their work shift. Since the worksite is remote, drivers are exposed to roads that are unpaved and at altitude (>1500 m) for up to two hours each way. Drivers’ work shifts are designed to accommodate the schedules of the workers such that, for example, drivers can deliver workers to the worksite before they start their shift (e.g., morning shift), and transport workers who are completing their shift (e.g., night shift) in a single round-trip. Drivers operate either large buses which transport up to 45 passengers per bus to the remote worksite or smaller buses up to 15 passengers, which shuttle employees within the remote worksite or provide concierge services for other employees.

### 2.2. Focus Groups with Drivers

All drivers at the bus organization were eligible to participate in a focus group, regardless of hours worked, tenure, or other factors. Drivers were recruited into the focus groups via a sign-up process. Six focus groups with 6–9 drivers in each group were held; three of the focus groups were completed with drivers of the large passenger buses and three were completed with drivers of the small passenger buses. Focus groups were held in-person and conducted by an experienced qualitative researcher and focus group moderator following a structured focus group guide. The focus groups were conducted in Spanish. Questions were structured into four main sections focusing on (1) drivers’ overall perspectives of their work, and their health, safety, and well-being; (2) specific outcomes of interest to the research team including job satisfaction and happiness, general health, absenteeism from work, job stress, musculoskeletal pain and injuries at work, and fatigue; (3) drivers’ perspectives of working conditions that influenced their safety, health, and well-being and drivers’ perceived priority areas; and, (4) barriers and facilitators to implementation of an organizational program to improve drivers’ safety, health, and well-being.

Questions were designed to answer each research question by probing and asking about information in different ways (Table 1). For example, working conditions influencing drivers’ safety, health, and well-being were captured by probing on how their work impacted various health and well-being concerns, as well as by asking questions directly related to their conditions of work. Each focus group took about an hour to complete.

### 2.3. Key Informant Interviews

Semi-structured key informant interviews were conducted with company personnel who held key roles in the organization with respect to workers’ health and safety or who had the power to enact change within the organization required for the implementation of the later intervention. These individuals held either management positions within the company or were union directors who represented the workers. In collaboration with the organization, researchers identified eight key informants to be interviewed. These included the company leadership, operations manager, safety managers, supervisors of the bus drivers, union directors, and a worker representative who participated in the company’s safety meetings. Interviews with key informants were conducted in-person by the same moderator who completed the focus groups, and were conducted in Spanish. Questions were structured into similar topic areas as the focus groups (Table 1).

### 2.4. Data Analysis

**Figure 1:**
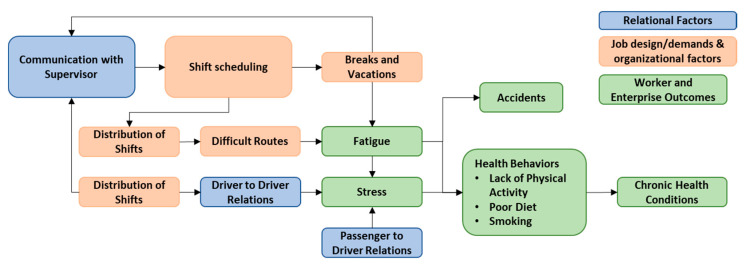
Map of Themes from Interviews and Focus Groups. Conditions of work captured by the relational (Blue), job demands, and organizational factors (Orange). Worker and Enterprise outcomes (Green) include the immediate proximal outcomes and the more distal downstream outcomes of accidents, health behavior, and chronic health issues.

The interviews and focus groups were conducted, audiotaped, and transcribed in Spanish. The transcriptions were then translated into English. The English transcripts were coded using NVivo software (Version 11, QSR International) and were then thematically analyzed [37]. We used a multi-stage coding process that included both predefined and emergent codes in a two-step process: we used a pre-defined coding tree based on the Harvard Center for Work, Health, and Well-being’s conceptual model [8]. New codes were added inductively to the coding tree as new themes arose in the data. Codes were structured in the coding tree under the following ‘parent codes’ which included: working conditions (e.g., shifts, scheduling, vacations, routes, compensation, benefits, physical environment, psychosocial work environment, promotions), job demands (e.g., alertness, workload, sedentary work), utilization of company resources and benefits (e.g., gym), safety (e.g., practices, regulations), worker health and well-being (e.g., mental health, stress, diet, chronic health conditions, health behaviors, fatigue/sleep), workers’ life outside of work (e.g., financial stressors, second jobs), enterprise outcomes (e.g., absenteeism, tardiness). Some of these codes also had additional child (sub-)codes. For example, child codes in the ‘psychosocial work environment’ included: supervisor support, work–life/family balance, violence and harassment, relationships with colleagues, relationships with passengers, and communication. Relationships between codes were then mapped using the ‘relationships function’ in NVivo. This function in the more recent version 11 of NVivo allows the coding of a special type of ‘relationship node’ that provides a connection between two codes [38]. These relationships between codes can then be displayed visually using the relationship mapping function, in NVivo. For example, a relationship between specific working conditions and health outcomes can be mapped (e.g., shift schedules impact the distribution of shift; the distribution of shifts can result in some drivers being required to drive the more difficult routes which take longer to complete, more often; this can then result in driver fatigue due to limited time to rest and sleep; fatigued drivers have limited time to exercise and tend to select pre-packaged or fast food options; these poor health behaviors were reported to result in higher incidences of chronic health conditions). We used this relationship map–generated from NVivo–to create a map of themes (as displayed in Figure 1). From these data, we developed a model linking working conditions and other organizational factors with drivers’ safety, health, and well-being.

## 3. Results

The analysis of the key informant interviews and focus groups resulted in overarching themes related to (a) working conditions—work relationships (supervisors, coworkers, and passengers), and organization of work (shifts, scheduling, and routes) and (b) health and well-being outcomes (fatigue, stress, health behaviors, chronic health conditions).

### 3.1. Work Relationships

#### 3.1.1. Driver Relationships with Supervisors

Drivers and supervisors felt differently with respect to their relationship with each other: drivers generally felt that they had a poor relationship with their supervisors, whereas supervisors considered their relationships with drivers had improved in recent times. This tension was noted by some of the key informants who operated at higher management levels within the company. Drivers stated that it was difficult communicating with their supervisors, notably when reporting using the emergency “stop work” code when drivers identified hazardous conditions. This code could be used to indicate if there were poor road conditions, the driver was feeling tired, or any other emergency that the driver was experiencing while on the road, and if called, another driver may even be called to take over their shift. Regarding utilizing this emergency code, a driver stated:

*“The person (supervisors) does not even take his time to ask, for example, what happened to you, whether you are OK, to show concern, you’d better go home…you, see? Right away, the first thing, a grim face. …You (the driver) have to apologize, to say the least, because you are not in good condition, when it should be the other way around.” (Driver)*

The shame of using the emergency code caused drivers to reconsider using the emergency code in the future. This led to an erosion of trust between the drivers and supervisors, something that the supervisors had been acutely aware of. Supervisors knew that drivers had feared retaliation due to reporting their concerns in the past. The supervisors had tried to improve the trust with their drivers, with one key informant stating:

*“We are supporting the drivers, helping them, we have even communicated to them that if it is necessary for a service to arrive late, we are going to arrive late, the important thing is not to have accidents, not to put the driver or the passengers at risk.” (Key Informant)*

Both drivers and supervisors stated that one of their main goals was to prevent drivers from driving tired, in unsafe conditions, or in an emergency. However, the lack of trust that the drivers had with the supervisors when reporting such situations inhibited effective communication between them. Supervisors and key informants both considered building this trust with their drivers as an important organizational goal that was currently not being adequately addressed. One key informant stated:

*“Something is still lacking; we have to continue to work in building a climate of trust.” (Key Informant)*

#### 3.1.2. Relationships between Drivers

Drivers, with few exceptions, thought positively of one another. Despite the drivers working in isolation day-to-day, many considered their fellow drivers to be more than just coworkers; some considered them to be friends and there was a certain level of comradery among them:

*“What I enjoy the most is comradeship. Here we share with our mates, we get together with different workmates, different people… it is very pleasant.” (Driver)*

However, despite this comradery, some drivers who had different job titles did feel some resentment due to their differing job demands. Small bus drivers stated that they must make additional stops beyond their schedule to pick up and drop off passengers. When this occurs, the small bus drivers do not receive additional compensation, but large bus drivers had previously fought to receive additional compensation for similar circumstances and now receive it. The small drivers failed to receive additional compensation after requesting it, leading these drivers to believe that they were of unequal standing within the company compared to the large bus drivers. Ultimately, this impacted the small bus drivers’ relationships with the large bus drivers and their supervisors. A small bus driver said the following:

*“We are not considered at all. Not even when we’ve had meetings with them (supervisors), you can’t talk, you can’t say anything.” (Driver)*

#### 3.1.3. Drivers’ Relationships with Passengers

Although drivers reported having generally good relationships with their passengers on a day-to-day basis, certain circumstances (such as passengers making special requests of the drivers), which occurred with regular frequency, caused additional challenges. This resulted in significant stress and additional pressures for the drivers. For example, drivers preferred cold air conditioning as it helped them stay awake during long shifts, but passengers would complain, requesting warm air as this helped them to sleep on their journey home after a long shift. Similarly, passengers often wished to be dropped off at a non-designated stop closer to their home, rather than at the authorized stop. This could result in drivers making many undesignated stops in a shift and adding considerable time to an already long shift for the driver. Drivers felt uncomfortable dealing with these situations and being conflicted with appeasing the passengers and keeping the client company happy (avoiding complaints being made). Drivers reported that they were often disrespected if they did not comply with passenger requests:

*“(The passengers) show the driver bad manners… lack of respect, say, lack of judgement toward the driver… that’s stressful. Us drivers have a huge responsibility in the end.” (Driver)*

### 3.2. Organization of Work

#### 3.2.1. Breaks and Vacation Time

Drivers were provided with vacation time by their organization; however, drivers stated that they had few opportunities to use it. The drivers often attempted to use their vacation time by making a request many months in advance; however, it could be canceled on short notice due to the scheduling needs of their supervisors. Drivers stated that this resulted in their often using their sick leave to take a vacation:

*“My wife works, and we never have vacations together. When she is on vacation I am working. And she says hey ’get’” a sick leave so that we have some days off. That is where sick leaves come from.” (Driver)*

Many drivers considered the use of sick leave as their only method to gain sufficient rest or take vacations. As more people used sick leave for vacations, fewer drivers could take their preplanned vacations. The company leadership stated that they were aware of this issue, but it had proven to be difficult to solve. A member of the leadership team reported that at any given time, nearly 10% of all drivers were out on sick leave, though it was unclear how many were using it for vacation purposes.

*“We have a severe problem that we’ve had a lot of demand for transport, and we’ve had to cut into the drivers’ vacation time, so we’re not giving them all the vacations that we ought to, so the vacation time starts to build up, the rest time that the drivers ought to have, and we’re postponing their vacations.” (Key Informant)*

#### 3.2.2. Distribution of Shifts

The frequency and distribution of shifts were the main concern discussed among the drivers. They acknowledged that they work irregular hours but that the scheduling can often be inflexible with regard to their personal and self-care needs. Additionally, there may only be a few hours rest for a driver between routes, depending on their route or shift allocation. Drivers may also need to work these shifts back-to-back, multiple days in a row. In some cases, workers reported only getting a few hours of sleep each night between shifts and this could occur over consecutive days. This leads to exhaustion, which can result in additional sick leave, and worry that if they do drive tired then they may be involved in an accident.

*“You get home by midnight, and you are supposed to get up at 4:30 a.m.… Then, you come here [to work] already feeling sleepy since you have slept just four hours… Then repeat the process 5 days in a row.” (Driver)*

#### 3.2.3. Route Scheduling

Of particular concern were the shift distribution and scheduling of a particularly challenging and difficult route (“Desafiante route”, name changed for anonymity). The Desafiante route was noted by nearly all drivers and key informants, and was much longer, had narrow and often congested roads, and intersected with areas with high traffic density.

*“The drivers say to themselves ‘I’m going down that route again,’ Now I’m going down in a bad mood, I’m going to be late, I’m going to run into a traffic jam…(Key Informant)”*

The way the Desafiante route was scheduled allowed the drivers to have only a few hours of rest if they were assigned it multiple days in a row. The added stress of workers asking for additional stops could increase the duration of the shift even longer. One driver thought that balancing the Desafiante route with other shorter, or simpler routes might make it easier to manage for the drivers and allow for more rest between shifts.

*“At least they should be distributed in a more balanced way. If I have a busy day, the next day I should have a more relaxing day, and the one who got a less busy day, the next day should have a busier day. It can be compensated in that way.” (Driver)*

However, those involved in scheduling in the company, and other key informants, explained that this was a very complex and dynamic problem, with shift schedules being provided by the client and changing rapidly. Compounded with an older scheduling system that did not accommodate the idiosyncrasies of their scheduling or shift needs and a workforce of approximately 450 drivers, it was an extremely difficult problem to solve.

#### 3.2.4. Bus Design and Maintenance

While the drivers acknowledged that the company had strong bus maintenance policies and practices in place, they highlighted a few aspects that negatively contributed to their safety, health, and well-being. Some drivers discussed issues associated with driving buses which are normally driven by other drivers. Since these buses are adjusted for the previous bus driver, the orientation of the seat is sometimes not in an optimal position, which leads to musculoskeletal pain. The drivers explained that the set-up cannot be easily changed before a shift and therefore one drives with the bus adjusted to another person’s requirements.

In addition, bus maintenance is occasionally not fully completed at the end of a late shift. In these instances, a driver may arrive for their shift the next day unaware that the bus was not completely serviced the night before, and this delays the start of the day. This can also result in some resentment between the drivers.

Drivers also noted problems with certain aspects of the buses that they perceived directly caused them pain and discomfort. For example, in many buses, the clutch was difficult to use. One driver described a significant injury he sustained as a result:

*“Yeah, the gearshift of the vehicles, the ones we drive, they are hell, shifting gears, and look, I’m not exaggerating, once I put the vehicle into reverse and ended up with my hand cut open.” (Driver)*

### 3.3. Driver’s Health and Well-Being

#### 3.3.1. Fatigue

Fatigue was the most frequently discussed safety concern for the drivers, supervisors, and other key informants interviewed. This is of concern because fatigue can result in accidents and in the worst cases, fatalities of both drivers and passengers in more serious crashes. While fatigue is a serious issue for professional drivers in general, in this scenario it manifested through trust concerns between drivers and supervisors. The difficulty, or reluctance, drivers had using vacation time or emergency codes meant that they had little ability to rest when needed. Fatigue compromised the health of drivers, as one driver explained:

*“Fatigue … makes your health poor, you lack sleep, it’s quite powerful, as time goes by you simply start wearing away.” (Driver)*

Drivers and informants also associated drivers’ fatigue with poor health behaviors and other chronic health conditions.

#### 3.3.2. Stress

Drivers discussed how stress arose from fatigue, stemming from a lack of rest, poor interactions with passengers, and the difficulty associated with driving certain routes. Individually, these factors might not be as significant; however, drivers emphasized that they were being impacted by these factors every day.

*“You really feel under pressure as you know that you have to get somewhere and the guy is gonna be worried about this, about that, about the guy over here, about the vehicle over here, I think all that makes you… I think all that stuff has a negative impact on your health.” (Driver)*

#### 3.3.3. Health Behaviors

In response to fatigue and stress, drivers stated that they coped by participating in unhealthy behaviors such as smoking, eating unhealthy foods, and drinking coffee in excess. Coffee, sugary and other high caloric foods, and smoking were used to stay awake while the drivers were operating on only a few hours of sleep.

*“You eat badly, you get used to eating badly or drinking lots of coffee. Some guys drink more, they start smoking a lot, drinking too much coffee to stay awake.” (Driver)*

Processed and ready-to-eat packaged foods were eaten because they allowed the drivers to rest for longer periods of time on their breaks.

*“Most of the sick leave is, some for stress, panic attacks, what else do we have…stomach aches, which are caused by the same (stress-related) eating disorders, excess sometimes of coffee, cigarettes. Basically, poor habits.” (Key Informant)*

#### 3.3.4. Chronic Health Conditions

Unhealthy behaviors, fatigue, and stress, and challenging working conditionswere perceived by both drivers and key informants to contribute to chronic health conditions such as diabetes, hypertension, and obesity. Drivers were aware that these chronic diseases were common among themselves and their coworkers, and considered their sedentary work environment, schedules, and diet choices as the primary causes.

*“[Our] diet messes you up… diabetes and hypertension are quite common among colleagues and it must be a result of that (not having time to eat)… we eat in a rush and not in an organized way.” (Driver)*

The bussing organization had been acutely aware of the issue of chronic disease amongst its drivers. It had implemented voluntary access to a gym, complimentary health checkups every six months to assess risks for chronic diseases, and access to a physiotherapist and a nutritionist. However, these benefits were reportedly under-utilized by drivers, possibly due to the fatigue that they were experiencing, and lack of time and energy to use these services.

*“We are constantly worrying about [the drivers], obesity is a common result of sedentary work, therefore we are always concerned about hypertension, high blood-pressure, obesity.” (Key Informant)*

### 3.4. Informing a Total Worker Health^®^ Intervention 

Grounded in the qualitative data, we developed a conceptualization by mapping how these themes were related (Figure 1). Working conditions were associated with drivers’ health and well-being outcomes including stress and fatigue and their health behaviors. These in turn were thought to impact more distally on drivers’ health conditions, such as obesity, diabetes, and hypertension, as well as contributing to drivers’ absences from work. This framework will be used to inform the intervention targets of a participatory *TWH* intervention with the same company.

## 4. Discussion

This study aimed to determine the factors which contributed to the safety, health, and well-being of professional bus drivers using a qualitative approach. Focus groups with drivers and interviews with key informants highlighted root causes through the conditions of work that were contributing to drivers’ safety, health, and well-being.

### 4.1. Working Conditions Influencing Bus Drivers’ Safety, Health, and Well-Being

The relationship that a worker has with their supervisor has been linked to workers’ well-being [39]. In this study, we also found that lack of trust and poor communication could have significant safety implications if drivers were reluctant to use “stop-work” emergency codes when needed. While the association between supervisor support and workers’ health and well-being has been well established in other sectors [40,41], little research has been conducted in transportation on psychosocial work environment factors. One prior study found that strong connections between drivers and dispatchers resulted in more efficient operations, and mutual respect between drivers and dispatchers often improved attitudes and driver retention [42].

Schedules and shifts have been well-established as being potentially harmful to workers’ health and well-being [43,44,45]. Workers with little control over their schedules and shifts have been found to have lower levels of subjective health than those with more control [46]. Longer working hours and short sleep duration (<6 h per day) have been found to be associated with various health conditions, such as metabolic and cardiovascular diagnoses [47]. In companies, such as the one in our study, where drivers are salaried, long working hours and few hours available to sleep between shifts are common and can be detrimental for both physical and mental health [48,49]. In our study, drivers were advised of their schedules at short notice with little control, resulting in considerable impacts on their ability to manage their work demands while maintaining healthy behaviors. Compounded with a growing workforce, and lack of technological support to assist with complex scheduling and shifts, this working condition can have potentially profound impacts on drivers’ fatigue, their health behaviors, and long-term health and well-being. As society continues to move towards a 24/7 approach to providing services, the need for work schedules characterized by shift work, rotating schedules, and longer working hours is also increasing; the future of work is likely to exacerbate these problems unless these working conditions are addressed.

There is a body of literature that supports the importance of the physical environment of buses as the main cause of musculoskeletal injuries and accidents [50,51]. This can also be influenced by external factors, such as the weather or road conditions. However, in our study, this was not identified as a main working condition influencing drivers’ safety, health, and well-being. One possible reason for this is the company’s strong focus on safety and ergonomics; in collaborating with this company, we observed mature safety systems, policies, and practices. The concerns highlighted by drivers and key informants could be related to issues in scheduling, for example, maintenance not occurring if a driver returns late from a shift, or last-minute changes resulting in another driver needing to use a bus not ergonomically set up for themselves.

### 4.2. Safety, Health, and Well-Being of Professional Bus Drivers

The common concerns for the health of drivers were fatigue, stress, and chronic health conditions. Poor health behaviors were caused by both stress and fatigue as well as lack of access to healthier food options and inability to exercise because of short breaks and rest time. This is not a new finding; these health conditions experienced by the drivers have been well-documented in the literature in several epidemiological studies of drivers’ safety and health [22,52]. What is new is the qualitative documentation of the lived experience of these safety, health, and well-being sequelae as a result of their exposures at work and the identification of the pathways and conceptual model (Figure 1). We found that drivers’ poor health behaviors had complex pathways through both the conditions of work and other health outcomes, driven by several factors in their work environment and compounded by fatigue and stress. Providing a solution to improve the health behavior directly—through healthier food options or providing access to physical exercise opportunities as has been found successful in other intervention studies for bus drivers—had been trialed in this company without success. For example, the company had attempted to address the obesity problem, and the issue of drivers not performing enough physical activity during the day, by providing them with an on-site fully equipped gym with a trainer, but, due to schedules, drivers did not have time or were too tired between shifts to use the gym. This proposed solution does not address the conditions of work.

### 4.3. Using Qualitative DATA to Inform Intervention Design

The findings from this study will be used to inform the content of a participatory *TWH* organizational intervention for bus drivers. Tailoring the intervention to fit the organization is important for implementation success because working conditions can manifest in different ways even within the same industry. Focusing on areas of priority for both the company leadership and, perhaps more importantly, the front-line workers, also contributes to implementation success. Demonstrating the importance of organizational fit, workers and company leaders are more likely to support and participate in program activities for areas that they believe are important priorities. Although legacy working conditions like the physical work environment and chemical hazards such as fumes are important exposures to consider, psychosocial and work organizational exposures are inherently complex and may require more in-depth exploration. This emphasizes the importance of qualitative, stakeholder-driven methods. Every organization is unique even within the same industry. To tailor an intervention that improves the safety, health, and well-being of drivers, we used qualitative methods, including focus groups and interviews, to provide valuable data to inform the intervention’s content. However, other methods such as workshops, ethnography, or mixed methods may also be useful depending on the context, time, and resources available to conduct this type of formative research.

### 4.4. Strengths and Limitations

A strength of this study is the participatory qualitative approach to identifying intervention content based on methods that we have used previously [28,29], and thus, is largely replicable. Through these methods, we obtained perspectives from key stakeholders within the organization including drivers (workers), union representatives and key decision makers (management). We tailored these methods so that they were feasible and easily implemented by an organization in a population of workers that are distributed away from a central workplace—in this case, being on-the-road driving buses or out in the field supporting operations, and safety and health. For example, focus groups were held on work time with drivers, and managers were interviewed at a time of their choosing.

The specific results and findings of this study may not be generalizable due to the study’s specific context and geographical location. Additionally, the organization was very supportive of the study and motivated to implement a *TWH* approach. They were amenable to providing organization time to facilitate the completion of the study. There was also significant support and commitment from leadership to enhance the well-being of the drivers at the organization. Other organizations may not have this support and may not be able to collect data as easily. However, the main implications from this study are grounded in the process, the importance placed on psychosocial and work organizational conditions, and the emphasis placed on using this type of approach to identify how these working conditions manifest as the root causes of workers’ safety, health, and well-being.

### 4.5. Implications for Future Research and Practice

Prior research has demonstrated differences in work organization needs between different types of professional drivers [16]. Identification of these needs requires a careful approach to prevent missing important underlying issues by solely relying on those that may be identified through a literature review or survey research. Future interventions should consider a formative qualitative research process involving key stakeholders to ensure that the intervention is tailored to the needs of the organization, and its employees. The “Integrative and Dynamic Healthy Commercial Driving Paradigm” was developed in 2015 as a response to the ineffectiveness of current health and wellness programs. It highlights identifying upstream causes of driver health outcomes by including all stakeholders in the development of interventions, and preventing poor health outcomes at primary, secondary, and tertiary levels [17]. Programs such as these, which address both the unique work organization of the transportation industry and act to build trust between drivers and supervisors, should be promoted to improve the health and well-being of professional drivers.

The transportation industry can use these findings by ensuring collaboration occurs between supervisors, management, and drivers when implementing safety, health, and well-being initiatives. While there may be success in organizations implementing programs to improve driver well-being by themselves, it is also possible that a poorly tailored program will fail to improve driver well-being. Determining the root cause of drivers’ health outcomes through a combination of focus groups and key informant interviews can enable management to create driver safety, health and well-being programs that are beneficial and effective.

## 5. Conclusions

This study’s participatory approach found that while the health outcomes impacting bus drivers may not be unique to an organization, the working conditions which facilitate the health and well-being outcomes can be. Because of this, the determination of which working conditions are causing poor safety, health, and well-being outcomes can be difficult to ascertain. Collecting data through focus groups with drivers and interviews with other key stakeholders can allow for candid discussions about these working conditions and how they uniquely manifest within an organization, which can then be addressed. Bussing organizations and future research can use this TWH approach to determine which working conditions should be modified to improve the safety, health, and well-being of drivers.

## Figures and Tables

**Table 1:** Examples of questions for the focus groups and key informant interviews.

Topic Area	Focus Group Questions	Key Informant Questions
Workers’ perspectives of their work and key health, safety, and well-being outcomes	What do you enjoy most about your job? What do you enjoy least about your job? What are the main health and safety considerations that impact you on your job? What about your job contributes to your overall quality of life and well-being?	What do think the key priorities for safety of drivers are at <company>? Beyond safety, what other health and well-being priorities are important at <company>? What are the concerns for drivers with respect to their safety, health, and well-being?
Focus on specific safety, health, and well-being outcomes (job satisfaction and happiness, general health, absenteeism from work, job stress, musculoskeletal pain and injuries at work, and fatigue)	What contributes to your overall happiness at work? Do you think your general health is impacted by your job? How?What are some of the main reasons that people miss days at work? Are there things about your work that you find stressful? Do you think that stress affects your health and safety on the job? Are there parts of your body that ache/are painful that you think are caused by or made worse by your work? What aspects of your job contribute to your symptoms? How does your job affect how tired you get during the day?	Can you tell me more about how <outcome> impacts on drivers’ safety, health, and well-being?
Working conditions influencing safety, health, and well-being	Thinking about your job, what are the working conditions that most impact your: ‑ General health ‑ Well-being Are there working conditions that make you feel unsafe at work?	While there are various ways to promote safety, health, and well-being, we want you to think broadly about how work is organized and managed here at <company>-how does the work environment influence drivers’ safety, health, and well-being? We would like your unique perspective regarding systems already in place and how policies and practices support or promote drivers’ safety, health, and well-being. Specific questions on working conditions were asked, e.g., what causes stress on the job? Are there aspects of the physical work environment that impact the drivers’ safety, health, and well-being?
Perspectives on priority areas	From your perspective, what should be the top priority with respect to improving: ‑ Worker well-being/ quality of life ‑ Worker health ‑ Worker safety	What types of policies, programs and practices do you see as beneficial in improving drivers’ safety, health, and well-being?
Determine barriers and facilitators to intervention	What are the most important things to think about when trying to make positive changes in the workplace? What things could get in the way of making changes in your workplace?	Are there initiatives in place that could be built upon that you think could really make a difference? What do you see as the main challenges we might face in developing a program?

## Data Availability

Data are available from the corresponding authors on reasonable request.
